# Radiomics-based T-staging of hollow organ cancers

**DOI:** 10.3389/fonc.2023.1191519

**Published:** 2023-08-30

**Authors:** Dong Huang, Xiaopan Xu, Peng Du, Yuefei Feng, Xi Zhang, Hongbing Lu, Yang Liu

**Affiliations:** ^1^ School of Biomedical Engineering, Air Force Medical University, Shaanxi, China; ^2^ Shaanxi Provincial Key Laboratory of Bioelectromagnetic Detection and Intelligent Perception, Shaanxi, China

**Keywords:** radiomics, hollow organ cancer, segmentation, T-staging, feature-based methods, deep learning-based methods

## Abstract

Cancer growing in hollow organs has become a serious threat to human health. The accurate T-staging of hollow organ cancers is a major concern in the clinic. With the rapid development of medical imaging technologies, radiomics has become a reliable tool of T-staging. Due to similar growth characteristics of hollow organ cancers, radiomics studies of these cancers can be used as a common reference. In radiomics, feature-based and deep learning-based methods are two critical research focuses. Therefore, we review feature-based and deep learning-based T-staging methods in this paper. In conclusion, existing radiomics studies may underestimate the hollow organ wall during segmentation and the depth of invasion in staging. It is expected that this survey could provide promising directions for following research in this realm.

## 1 Introduction

Cancer occurrence in hollow organs, such as gastric cancer (GC), colorectal cancer (CRC), esophageal cancer (EC), cervical cancer (CC), and bladder cancer (BC), poses a significant threat to human health. Global cancer statistics in 2022 reveal that hollow organ cancers rank fifth and fourth among the top ten cancers in terms of incidence and mortality, respectively (1). Despite being inconspicuous and difficult to detect in their early stages, hollow organ cancers often advance to the late stages by the time symptoms manifest (2–6).

Early screening and diagnosis are crucial for reducing morbidity and mortality associated with these cancers due to their slow progression. For instance, the implementation of screening for CRC in the United States led to a reduction of over 40% in its incidence and mortality (7). Current screening and diagnostic methods include fecal-occult-blood screening (8), blood tests for tumor markers (9), optical endoscopy (10), and imaging tests (11). However, the low specificity of the former two methods renders their results only as reference points (12). Although optical endoscopy is the clinical gold standard for hollow organ cancer diagnosis, its invasive nature can result in organ damage, such as infections, perforations, and hemorrhages (13). Noninvasive imaging techniques, such as MRI, CT, and US, have shown significant potential for cancer screening and diagnosis (14).

For the diagnosis of hollow organ cancers, T-staging plays an integral role, significantly impacting treatment decisions and prognoses. For example, T2 or below-stage BC patients may undergo cystoscopic resection, while those with more advanced T-stages may require complete cystectomy (15). In the case of EC, the selection of radiotherapy and chemotherapy is determined by whether the tumor is beyond T2-stage (16). Similar considerations apply to other hollow organ cancers (17–19). Radiomics methods offer the ability to quantify tumors through the extraction of extensive features and evaluate tumor heterogeneity objectively (14), surpassing subjective interpretations by medical professionals. Consequently, imaging-based radiomics studies have gained attention from researchers.

Given their clinical significance, consolidating and mutually referencing individual studies on hollow organ cancers becomes necessary for accurate diagnosis. In this review, we systematically describe and compare feature-based and deep learning-based methods sequentially, based on their respective feature extraction techniques. By analyzing their strengths and limitations, we aim to provide a comprehensive overview of existing studies and propose corresponding insights and limitations. Ultimately, our review aims to guide downstream diagnosis of hollow organ cancers and inspire subsequent radiomics studies to address these limitations progressively, thus developing more effective solutions.

## 2 Review methodology

Peer-reviewed papers were collected primarily through online digital libraries such as PubMed, ScienceDirect, Springer and IEEE Xplore. In addition, the Google Scholar search engine was used to search for pertinent publications. Considering their high incidence and mortality, we used the five following hollow organ cancers as examples: GC, CRC, EC, CC, and BC. For each disease, we mainly introduce their feature-based and deep learning-based T-staging methods. Therefore, the search keywords used included ‘radiomics’, ‘hollow organ cancer’, ‘segmentation’, ‘T-staging’, ‘gastric cancer’, ‘colorectal cancer’, ‘esophageal cancer’, ‘cervical cancer’ and ‘bladder cancer’. Only papers that included medical images were considered. The papers that were collected for review were published between 2012 and 2023, since imaging-based radiomics started receiving attention in 2012.

During the literature review, the following items were considered:

- Objective of the paper (learning tasks);
- Visual input (the kind of medical images);
- Methods used (segmentation methods, feature extraction methods, classification methods);
- Results or findings (the results obtained).

## 3 The pipeline of radiomics for T-staging

**Figure 1:**
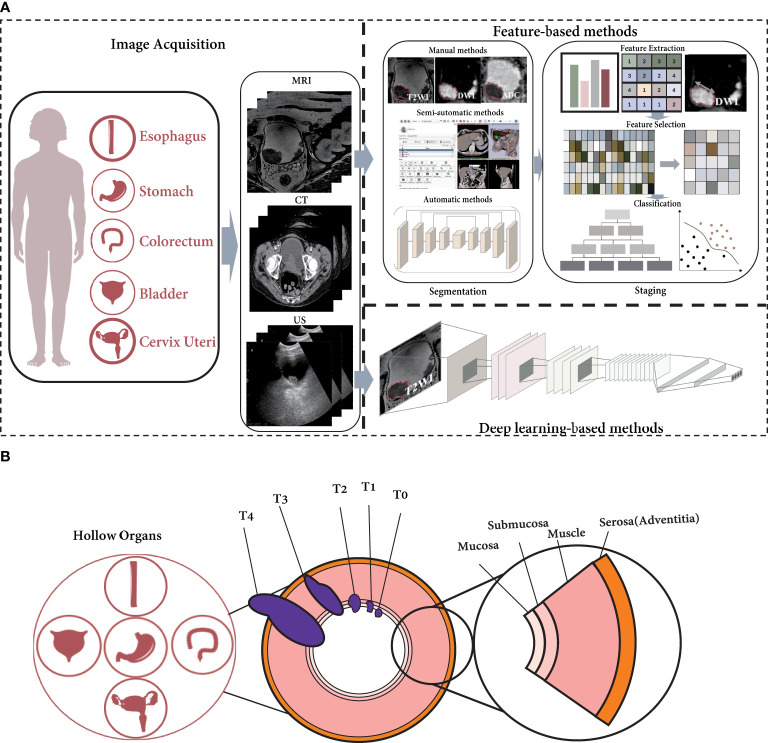
The current radiomics pipeline is not appropriate for hollow organ cancers with specific growth characteristics. **(A)** The pipeline of radiomics for T-staging. **(B)** The growth of cancer in hollow organs invade the organ walls.

The pipeline of radiomics for tumor staging is shown in **Figure 1A** (14, 20). In feature-based methods, segmentation is the most basic step. We introduce three different types of segmentation techniques: manual, semiautomatic, and automatic segmentation in this paper. For tumor staging, many radiomics studies are based on conventional handcrafted features, i.e., shape, texture, statistics, wavelets, etc. (15, 21) The features are then screened to reduce the risk of overfitting the classifier, after which the features are classified. This is the standard pipeline for feature-based radiomics. For deep learning-based algorithms, on the other hand, the original image is frequently used as input, and stage prediction is conducted directly through an end-to-end structure.

However, hollow organ tumors adhere to the inner surface of the hollow organ when they occur, invasively grow toward the outer surface, and metastasize to the lymph nodes, as shown in **Figure 1B**. According to the eighth edition of the American Joint Committee on Cancer (AJCC) Staging Manual (22, 23), the depth of the tumor-invasive hollow organ wall is an important criterion for T-staging. (The T-staging of hollow organ cancers are listed in **Table S1**) In addition, the manual recommends the use of imaging modalities for staging diagnosis (23). As a result, T-staging is often not only related to the tumor territory seen in imaging; the depth of invasion is also an important factor.

For feature-based methods, the first step is usually to segment the tumor region (14). Because in many cancers, tumor size is a key factor, as in breast cancer and hepatobiliary cancer (23–25). However, unlike these solid organ tumors, in hollow organ tumors, the depth of tumor invasion is an important indicator for staging (23, 26–28). Therefore, general radiomics approaches for the T-staging of solid organ cancers are not fully applicable to that of hollow organ cancers. The relationship between tumor stage and depth of invasion determines that the segmentation of organ wall is also important. In radiomics studies of hollow organ cancers, the tumor and organ wall should be segmented first, and then the tumor stage can be estimated.

For deep learning-based methods, since there is no need to segment the region of interest (ROI), the segmentation of tumor and organ wall is not necessary (29, 30). However, end-to-end structures are often disfavored for their inexplicability. Therefore, for hollow organ cancers, how to integrate the depth of tumor invasion into tumor stage as an auxiliary task may be an issue that researchers need to be considered.

## 4 Segmentation for feature-based methods

### 4.1 Manual methods

Regarding the segmentation of the ROI, in most radiomics studies, it is still necessary to invite professional medical staff to manually outline it. For instance, in Ba-Ssalamah et al. (31), a radiologist was invited to manually delineate the ROI. In Ahn et al. (32), the radiologist also asked for help from another individual to confirm the manual result, which ensured intra-observer agreement. In addition, in Liu et al. (33), two radiologists jointly segmented the ROI manually. Similarly, in Wu et al. (34), multiple people participated in manual delineation, but they invited additional experts to confirm the individual delineation results to ensure inter-observer consistency. Therefore, regardless of whether manual delineation involves a single person or multiple people, the consistency guarantee of the delineation needs to be emphasized.

### 4.2 Semiautomatic methods

A specific software can be used to aid in the segmentation of a target regions or volumes. Usually, the software reduces labor use based on the key points of manual drawing and the differences around the pixels. For instance, 3D slicer (35), and PMOD 3.6 (36) can be used to segment the ROI or volume of interest (VOI). There are also several semiautomatic methods that can be used by artificially setting thresholds or initial shapes, such as those described in Dong et al. (37); Mu et al. (38); Xu et al. (39). In these works, we also determined whether the surrounding tissue was considered, since these segmentation methods were applied in hollow organ cancers. According to our statistics, the surrounding tissues were also segmented in Shen et al. (40); Mu et al. (38). However, the researchers did not segment the organ wall. With their methods, the depth of invasion could not be fully represented, which may result in inaccurate downstream T-staging.

### 4.3 Automatic methods

Many researchers expect to find a completely automatic method. This kind of method does not require manual intervention and learns the required segmentation area through a deep network. In these works, various deep networks are employed, such as two-stream 3D CNNs (41), and U-Net (42), and progressive dilated CNNs (43). In these works, several researchers segmented the ROI of each slice from a 2D perspective, as in Lin et al. (42); Dolz et al. (43). Others intended to segment the VOI from a 3D perspective (41, 44). With 2D methods, it is necessary to comprehensively analyze the multi-slice segmentation results to obtain the depth of invasion for T-staging. With 3D methods, it is more straightforward to estimate the depth of invasion by voxels.

From aforementioned statements, manual segmentation or semiautomatic segmentation using software is used as the gold standard, and the performance of these methods does not need to be considered; rather, quantitative metrics can be applied. For consistency, we suppose that it is necessary for manual and semiautomatic methods that require human involvement to ensure the accuracy of segmentation. Due to the lack of human intervention, fully automated methods do not require consistency checks. Since hollow organ tumor growth will appear to be infiltrative, we believe that the inclusion of the hollow wall is necessary to accurately estimate its invasion, as in the studies described in Liu et al. (45); Wang et al. (46). These items are summarized in **Table S2**.

## 5 Staging for feature-based methods

hollow organ cancers that have been staged via feature-based methods include EC (34, 47), GC (33, 48, 49), CRC (17, 21), CC (38), and BC (15, 46, 50). In these works, GC and BC were more commonly staged. The reason may be that the volume of the wall tissue in these two organs is relatively empty compared to that of the tumor, and the tumor and the wall tissue can be clearly distinguished by imaging. The esophagus and cervix are relatively narrow, and there are many folds in the colorectum, making the tumor and organ wall less obvious on imaging.

When staging a tumor, most studies try to distinguish tumors less advanced or more advanced than stage II, e.g., Liang et al. (17); Liu et al. (48); Wu et al. (34); Mu et al. (38); Wang et al. (49). The main reason is that the two types of treatment methods are different. Resection is regarded as the most common treatment option for patients with a tumor stage less advanced than T2, while chemotherapy is generally the main treatment option for patients with a tumor stage more advanced than T2 (51). For BC studies, researchers are more concerned about local excision surgery or total excision surgery, so more detailed T2 staging results is necessary (nonmuscle invasive vs. muscle invasive).

Concerning imaging studies, different studies involve various modalities. As a convenient and accessible imaging modality, CT is widely used, as in Liang et al. (17); Li et al. (21); Wu et al. (34); Wang et al. (49). Diffusion-weighted imaging (DWI) is another imaging modality that is commonly used (33, 48). Some studies have described the use of PET (47) or PET/CT (38) for T-staging. In addition, several studies have attempted to analyze tumor stage from multiple perspectives using multimodal MRI (15, 46, 50). Usually, the use of images is related to the data acquisition method and the type of disease. When predicting tumor stage in hollow organ, under certain conditions, it is better to use higher quality images and more modalities to show the invasion depth.

In these methods, radiomics features are the most utilized, e.g., Xu et al. (15); Liang et al. (17); Li et al. (21); Wu et al. (34); Ma et al. (47); Wang et al. (49); Zhang et al. (50); Wang et al. (46). Other hand-crafted features are introduced in Liu et al. (33, 48); Mu et al. (38). These features are usually selected to find the most effective features by the LASSO method (21, 34). Based on these features, several statistical methods are used to analyze the relationship between features and tumor stage (17, 33, 34, 47, 48. In other studies, various classifiers, such as support vector machines (SVMs) (15, 21, 38, 50), random forests (49), and logistic regression (46), have been chosen to classify these features. The basic workflow of these feature-based radiomics studies for T-staging of hollow organ cancers is almost the same. Because the data sources are inconsistent, it is difficult to judge which methods perform better. However, these methods use only general radiomics features to represent tumors. As stated in Sec. 3, they may ignore the feature representation of the organ wall, which leads to the inability to determine the invasion depth (23).

## 6 Deep learning-based methods

Recently, with the development of deep learning technologies, this realm has achieved better performance than traditional handcrafted features in various fields. Therefore, radiomics based on deep learning has also received increasing attention.

Studies of EC (29), CRC (52), and BC (53) have used deep learning-based methods for T-staging. This type studies are relatively lacking, since doctors usually use optical endoscopy to screen polyps in hollow organs. Sufficient data are produced in screening, so the existing deep learning work prefers optical images, e.g., Pacal et al. (54); Zhang et al. (55). However, for patients with hollow organ cancer, T-staging is also crucial and directly determines the treatment method. Therefore, research on deep learning-based methods using imaging for T-staging is still needed.

These deep learning works use imaging modalities such as PET (29), CT (52), and T2-weighted imaging (T2WI) (53). Deep learning usually requires a large amount of data for training. However, the data available in these studies are still insufficient (at most, more than 100 cases), so researchers have adopted several techniques that can reduce the risk of overfitting, such as using pretrained models (29), adding additional tasks (52), and using clinical rules (53) to constrain the training of deep networks. Therefore, currently, it may be necessary to construct a large-scale public database of hollow organ cancers for deep learning. This will greatly facilitate the application of deep learning in T-staging.

Several conventional deep networks, such as VGG (29), U-Net (52), and ResNet (53), are introduced in these studies. However, these end-to-end approaches may be too general. Since T-staging requires more attention to the mixed areas of the tumor and hollow organ wall, determining the invasion depth may be a better solution. For instance, in Zhang et al. (53), the authors proposed a two-branch architecture to predict the tumor stage of BC. In one branch, they took the tumor growth volume inside the bladder as a clinical rule to constrain stage prediction. In fact, their work considered the depth of tumor invasion into the bladder wall to some extent. Therefore, adding an additional task of consideration the invasion depth to the current end-to-end network may be more targeted.

In general, deep learning radiomics work is still relatively lacking, mainly because staging is not like segmentation, where multiple slices of images from each case can be used as training samples. However, it can be concluded from studies of other problems in tumors [e.g., Gao et al. (56); Bhatla et al. (57)] that deep learning may be a better tool for T-staging since features learned by deep networks are more representative than handcrafted features. Therefore, considering deep learning methods is a better choice when there is sufficient data. In addition, existing methods [e.g., Liang et al. (17); Mu et al. (38); Zhang et al. (50); Wang et al. (46)] mostly predict tumor stage based on tumor region and fail to consider the key factor in the definition of T-staging for hollow organ cancer, i.e., the depth of tumor invasion. Targeted feature extraction of this factor could more accurately represent tumor stage.

## 7 Discussion

Hollow organ tumors have garnered considerable attention in research, and this paper focuses on reviewing feature-based and deep learning-based T-staging studies across five representative hollow organ cancers. To provide clarity, we have divided our discussion into separate sections for feature-based radiomics and deep learning-based methods. While these studies have made remarkable progress in realizing automatic T-staging for hollow organ cancers, several limitations persist.

Regarding feature-based radiomics, three segmentation methods have been commonly employed, with manual segmentation being the gold standard but costly and time-consuming. Although semiautomatic methods offer some relief, ensuring their consistency remains a challenge. Furthermore, these methods face difficulty in overall characterization of the hollow organ tumor or wall, as they primarily rely on pixel gradient information. Fully automatic methods show promise in addressing these concerns, but their accuracy still requires improvement due to limited medical image data availability. Additionally, we emphasize the importance of considering hollow organ wall segmentation, as wall tissue invasion serves as a critical reference for downstream T-staging. Unfortunately, most existing research does not prioritize this aspect.

**Figure 2:**
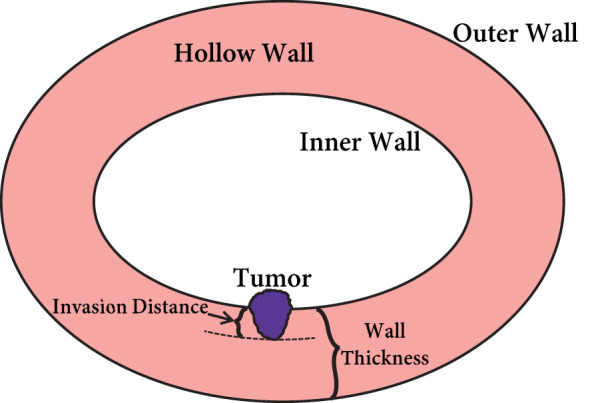
The depth of tumor invasion in the hollow organ wall.

In the context of feature-based T-staging, the current radiomics pipeline favors solid organs over hollow organs, as it disregards the organ wall’s role. The depth of tumor invasion holds significant discriminative information for T-staging in hollow organ cancers, but few studies have quantified this parameter. We propose that subsequent studies should prioritize quantifying tumor invasion depth to explain algorithmic decision-making to physicians. As depicted in **Figure 2**, tumor invasion depth is determined by the ratio of invasion distance to wall thickness: *InvasionDepth = InvasionDistance/WallThickness*. Hence, segmenting both the tumor and organ wall is crucial for radiomics approaches in hollow organ cancer T-staging.

Deep learning-based T-staging requires the availability of more public data for improved performance. Moreover, end-to-end approaches may overlook the significance of segmenting the ROI or VOI. To incorporate the advantages of feature-based approaches, it may be more effective to include an additional segmentation task for tumor and organ wall segmentation in deep learning-based methods. By calculating the depth of invasion based on these segmentation results, a more refined T-staging method could be achieved.

## 8 Conclusions

Hollow organ cancers have always been at the forefront of cancer prevalence and mortality due to their special structures. Radiomics, a noninvasive approach, has become the most effective and convenient way to diagnose these tumors. This paper reviews T-staging related research on two kinds of radiomics methods: feature-based and deep learning-based. To segment hollow organ tumors, the feature-based methods discussed in this review include manual, semiautomatic, and automatic segmentation. In addition, we present the findings of feature-based and deep learning-based T-staging studies for various hollow organ cancers. While acknowledging the progress made by existing studies, it is important to recognize the limitations of feature-based and deep learning-based methods. In future research, it is essential to develop a specialized framework for radiomics in hollow organ cancers, differentiating them from solid organ cancers. This framework should consider the unique growth characteristics of hollow organ tumors. Regardless of the methodology employed, whether feature-based or deep learning-based, quantifying the depth of tumor invasion into the organ wall emerges as a crucial indicator for T-staging.

## Author contributions

DH drafted this manuscript. XX helped perform the analysis with constructive discussions. XZ helped revise this manuscript. PD and YF helped to collect relevant literature. HL contributed significantly to manuscript preparation. YL contributed to the main idea of this review. All authors contributed to the article and approved the submitted version.
