# Supplementary material for: Radiomics-based T-staging of hollow organ cancers
Source: Front Oncol. 2023 Aug 30;13:1191519. doi: 10.3389/fonc.2023.1191519 (PMC10499612; doi:10.3389/fonc.2023.1191519)
Supplement: Supplementary file 1 [file Table_1.pdf]

frontiers

Supplementary Material

1 SUPPLEMENTARY TABLES AND FIGURES

As shown in Table S1, the tumor stages of hollow organ cancers are listed (according to the eighth edition of the American Joint Committee on Cancer (AJCC) Staging Manual) Amin et al. (2017b,a). Notably, for CC, the commonly used Federation of Gynecology and Obstetrics (FIGO) stage is also given. An important criterion when defining the tumor stage is the depth of the tumor-invasive hollow organ wall. In addition, the manual recommends the use of imaging modalities for staging diagnosis Amin et al. (2017b). As a result, the staging of these tumors is often not only related to the tumor territory seen in imaging; the depth of invasion is also an important factor.

1.1 Tables

Table S1. The T-staging of hollow organ cancers

EC	GC	CRC	CC (FIGO)	BC
T1: Tumor invades the lamina propria, muscularis mucosae, or submucosa.	T1: Tumor invades the lamina propria and, muscularis mucosae, or submucosa.	T1: Tumor into (but not through) submucosa.	T1 (I): The tumor is strictly confined to the cervix.	T1: Tumor invades subepithelial connective tissue.
T2: Tumor invades the muscularis propria.	T2: Tumor invades muscularis propria.	T2: Tumor into (but not through) muscularis propria.	T2 (II): The tumor invades beyond the uterus, but has not extended into the lower third of the vagina or to the pelvic wall.	T2: Tumor invades muscularis propria.
T3: Tumor invades adventitia.	T3: Tumor penetrates the subserosal connective tissue without invasion of the visceral peritoneum or adjacent structures.	T3: Tumor through muscularis propria into subserosa, or into non-peritonealised pericolic/perirectal tissues.	T3 (III): The carcinoma involves the lower third of the vagina and/or extends to the pelvic wall and/or causes hydronephrosis or nonfunctioning kidney and/or involves pelvic and/or para-aortic lymph nodes.	T3: Tumor invades perivesical tissue.
T4: Tumor invades adjacent structures.	T4: Tumor invades serosa or adjacent structures.	T4: penetration of the visceral peritoneal layer or adhesion to adjacent organs.	T4 (IV): The carcinoma has extended beyond the true pelvis or has involved the mucosa of the bladder or rectum.	T4: Tumor invades: prostatic stroma, seminal vesicles, uterus, vagina, pelvic wall, abdominal wall.

From Table S2, manual segmentation or semiautomatic segmentation using software is used as the gold standard, and the performance of these methods does not need to be considered; rather, quantitative metrics can be applied. For consistency, we suppose that it is necessary for manual and semiautomatic methods that require human involvement to ensure the accuracy of segmentation. Due to the lack of human intervention, fully automated methods do not require consistency checks. Since hollow organ tumor growth will appear to be infiltrative, we believe that the inclusion of the hollow wall is necessary to accurately estimate its invasion, as in the studies described in Liu et al. (2020); Wang et al. (2020). Therefore, we performed statistical analysis of these studies in which the wall was included. Furthermore, we wondered whether these segmentation methods were performed slice-by-slice or the entire 3D structure was segmented, as this factor might also be an aspect that affects the judgment of invasion depth.

Table S2. The segmentation for feature-based methods.

	References	Disease	Images (case)	Methods	Performance	Consistency Check	Wall Including	2D/3D
Manual	Ba-Ssalamah et al. (2013)	GC	CT (140)	The supervision of radiologists.	Not necessary.	53	✓	2D
	Ahn et al. (2016)	CRC	CT (235)	The measurement of one radiologist.	Not necessary.	✓	✓	2D
	Liu et al. (2018)	GC	DWI (64)	Manually drawn.	Not necessary.	53	✓	2D
	Wu et al. (2018)	EC	CT (154)	Manually outlined.	Not necessary.	✓	✓	2D
Semiautomatic	Ma et al. (2017)	GC	CT (40)	3D slicer software.	Not necessary.	✓	✓	3D
	Tsujikawa et al. (2017)	CC	PET/CT (83)	Manually outlined with PMOD 3.6.	Not necessary.	53	53	3D
	Dong et al. (2013)	EC	PET/CT (40)	Prior knowledge.	Not necessary.	53	53	2D
	Mu et al. (2015)	CC	PET/CT (42)	Combining the intensity and gradient information.	DSC 0.9178±0.0166 and Hausdorff distance 7.94±1.99.	53	✓	2D
	Xu et al. (2017)	BC	MRI (5)	Convex relaxation optimization.	DSC with 0.873.	53	✓	2D
Automatic	Jin et al. (2019)	EC	PET/CT (110)	Two-stream 3D CNN	The DSC is 0.764±0.134.	Not necessary.	✓	3D
	Lin et al. (2020)	CC	DWI (169)	U-Net	The DSC of 0.82 are obtained.	Not necessary.	53	2D
	Dolz et al. (2018)	BC	MRI (60)	Progressive dilated CNN	DSC of 0.98, 0.84, and 0.69 for inner wall, outer wall, and tumor.	Not necessary	✓	2D
	Rigaud et al. (2021)	CC	CT (247)	2D DeepLabV3+ and 3D U-Net	DSC 0.78 (2D model) and 0.79 (3D model).	Not necessary.	✓	Both

REFERENCES

Ahn, S. J., Kim, J. H., Park, S. J., and Han, J. K. (2016). Prediction of the therapeutic response after folfox and folfiri treatment for patients with liver metastasis from colorectal cancer using computerized ct texture analysis. *European journal of radiology* 85, 1867–1874

Amin, M. B., Greene, F. L., Edge, S. B., Compton, C. C., Gershenwald, J. E., Brookland, R. K., et al. (2017a). The eighth edition ajcc cancer staging manual: Continuing to build a bridge from a population-based to a more ”personalized” approach to cancer staging. *CA Cancer J Clin* 67, 93–99. doi:10.3322/caac.21388

Amin, M. B., on Cancer., A. J. C., and Society., A. C. (2017b). *AJCC cancer staging manual* (Chicago IL: American Joint Committee on Cancer, Springer), eight edition / editor-in-chief, mahul b. amin, md, fcap ; editors, stephen b. edge, md, facs and 16 others ; donna m. gress, rhit, ctr - technical editor ; laura r. meyer, capm - managing editor. edn.

Ba-Ssalamah, A., Muin, D., Schernthaner, R., Kulinna-Cosentini, C., Bastati, N., Stift, J., et al. (2013). Texture-based classification of different gastric tumors at contrast-enhanced ct. *European journal of radiology* 82, e537–e543

Dolz, J., Xu, X., Rony, J., Yuan, J., Liu, Y., Granger, E., et al. (2018). Multiregion segmentation of bladder cancer structures in mri with progressive dilated convolutional networks. *Medical physics* 45, 5482–5493

Dong, X., Xing, L., Wu, P., Fu, Z., Wan, H., Li, D., et al. (2013). Three-dimensional positron emission tomography image texture analysis of esophageal squamous cell carcinoma: relationship between tumor 18f-fluorodeoxyglucose uptake heterogeneity, maximum standardized uptake value, and tumor stage. *Nuclear medicine communications* 34, 40–46

Jin, D., Guo, D., Ho, T.-Y., Harrison, A. P., Xiao, J., Tseng, C.-K., et al. (2019). Accurate esophageal gross tumor volume segmentation in pet/ct using two-stream chained 3d deep network fusion. In *International Conference on Medical Image Computing and Computer-Assisted Intervention* (Springer), 182–191

Lin, Y.-C., Lin, C.-H., Lu, H.-Y., Chiang, H.-J., Wang, H.-K., Huang, Y.-T., et al. (2020). Deep learning for fully automated tumor segmentation and extraction of magnetic resonance radiomics features in cervical cancer. *European Radiology* 30, 1297–1305

Liu, S., Zheng, H., Zhang, Y., Chen, L., Guan, W., Guan, Y., et al. (2018). Whole-volume apparent diffusion coefficient-based entropy parameters for assessment of gastric cancer aggressiveness. *Journal of Magnetic Resonance Imaging* 47, 168–175

Liu, Y., Zheng, H., Xu, X., Zhang, X., Du, P., Liang, J., et al. (2020). The invasion depth measurement of bladder cancer using t2-weighted magnetic resonance imaging. *BioMedical Engineering OnLine* 19, 1–13

Ma, Z., Fang, M., Huang, Y., He, L., Chen, X., Liang, C., et al. (2017). Ct-based radiomics signature for differentiating borrmann type iv gastric cancer from primary gastric lymphoma. *European Journal of Radiology* 91, 142–147

Mu, W., Chen, Z., Liang, Y., Shen, W., Yang, F., Dai, R., et al. (2015). Staging of cervical cancer based on tumor heterogeneity characterized by texture features on 18f-fdg pet images. *Physics in Medicine & Biology* 60, 5123

Rigaud, B., Anderson, B. M., Zhiqian, H. Y., Gobeli, M., Cazoulat, G., Sderberg, J., et al. (2021). Automatic segmentation using deep learning to enable online dose optimization during adaptive radiation therapy of cervical cancer. *International Journal of Radiation Oncology* Biology* Physics* 109, 1096–1110

Tsujikawa, T., Rahman, T., Yamamoto, M., Yamada, S., Tsuyoshi, H., Kiyono, Y., et al. (2017). 18 f-fdg pet radiomics approaches: comparing and clustering features in cervical cancer. *Annals of nuclear medicine* 31, 678–685

Wang, H., Xu, X., Zhang, X., Liu, Y., Ouyang, L., Du, P., et al. (2020). Elaboration of a multisequence mri-based radiomics signature for the preoperative prediction of the muscle-invasive status of bladder cancer: a double-center study. *Eur Radiol* 30, 4816–4827. doi:10.1007/s00330-020-06796-8

Wu, L., Wang, C., Tan, X., Cheng, Z., Zhao, K., Yan, L., et al. (2018). Radiomics approach for preoperative identification of stages i- ii and iii- iv of esophageal cancer. *Chinese Journal of Cancer Research* 30, 396

Xu, X.-p., Zhang, X., Liu, Y., Tian, Q., Zhang, G.-p., Yang, Z.-y., et al. (2017). Simultaneous segmentation of multiple regions in 3d bladder mri by efficient convex optimization of coupled surfaces. In *International Conference on Image and Graphics* (Springer), 528–542
